# ADHD-related symptoms among adults in out-patient psychiatry and female prison inmates as compared with the general population

**DOI:** 10.3109/03009730903532333

**Published:** 2010-03-10

**Authors:** Dan Edvinsson, Kerstin Bingefors, Eva Lindström, Tommy Lewander

**Affiliations:** ^1^Department of Neuroscience/Psychiatry Ulleråker, Uppsala University Hospital, UppsalaSweden; ^2^Department of Pharmacy, Uppsala University, UppsalaSweden; ^3^Department of Clinical Science/Forensic Psychiatry, MASSweden

**Keywords:** ADHD, adult, co-morbidity, convict, gender, general population, prisoner, psychiatry

## Abstract

**Objective:**

To compare the prevalence of symptoms consistent with attention deficit hyperactivity disorder (ADHD) and related problems in adults in the general population, out-patient psychiatry (where females are in majority), and female convicts.

**Method:**

A questionnaire based on the DSM-IV criteria of ADHD, reported childhood symptoms, reading and spelling problems, difficulties and suffering, and general assessment of functioning (GAF) was distributed to samples of the general population, open care psychiatry, and female prison inmates. Completed questionnaires were received from 517/1000, 349/400, and 50/65 of the three samples, respectively.

**Results:**

Symptoms consistent with ADHD were more than three times higher in out-patient psychiatry than in the general population (6.6% versus 2.1%), with a male-to-female ratio of 1.6–1.7. The severity of symptoms and frequencies of associated disabilities were similar in men and women. ADHD symptoms and related problems occurred in 50% of the female prisoners, which is similar to male prisoners according to the literature.

**Conclusion:**

The high prevalence of symptoms and disabilities of ADHD in women should lead to awareness of the disorder in both sexes and be addressed in terms of diagnostic work-up, treatment, and rehabilitation.

## Introduction

According to the Diagnostic and Statistical Manual of Mental Disorders, 4th ed, text revision (DSM-IV-TR) (1), attention deficit hyperactivity disorder (ADHD) is characterized by a persistent pattern of inattention and hyperactivity-impulsivity with an onset before age 7. Three subtypes are described: the predominantly inattentive type, the predominantly hyperactive-impulsive type, and the combined type. The two latter subtypes correspond to hyperkinetic disorder (HKD) according to International and statistical classification of diseases and related health problems. Tenth revision (ICD)-10 (2). The symptoms must be present in at least two settings (home, school, or work) and cause impairment of social, academic, and/or occupational functioning. ADHD is considered to be the most common mental disorder in childhood and is estimated to affect about 3%–7% of school-aged children (1). A recent meta-analysis of 102 prevalence studies concluded that the overall prevalence of ADHD was 5.29% (3).

Follow-up studies of afflicted children into adulthood have shown persistence of syndromatic ADHD or ADHD in partial symptomatic remission with continued functional impairment in a majority of cases (4). The prevalence of adult ADHD has been estimated at 2.9%–4.4% (5,6). Barkley et al. (7) conclude that at least 5% of the US adult population suffer from ADHD and that it affects most aspects of life if persistent into adulthood. An epidemiological study covering several countries in Europe, the Middle East, and the Americas reported prevalence estimates of adult ADHD at 1.2%–7.3%, with an average of 3.4% (8). Thus, adult ADHD is among the most prevalent mental disorders.

Several reviews of adult ADHD have been published during recent years (7,9). The age of onset criterion, i.e. before age 7, is an issue under discussion (7,10). Patients may not have been diagnosed in childhood, or symptoms of ADHD may not become prominent or severe enough until adolescence, and therefore appear to have a later onset. Thus, they do not fulfil the DSM-IV-TR criteria for ADHD. Barkley et al. (7) have proposed that an age of onset during adolescence should be discussed for DSM-V.

Co-morbidity of ADHD with other mental and neuropsychiatric disorders is common (1) and has been reviewed and described by Barkley and Brown 2008 (11), Nylander et al. (12), and others. Thus, anxiety and affective disorders, especially bipolar disorder (13,14), alcohol and substance abuse and dependence (15), posttraumatic stress disorder (PTSD) (16), border-line personality disorder (17), often co-occur with ADHD. There is also a high prevalence of learning disabilities (18,19) and reading difficulties among children, adolescents, and adults with ADHD (20,21). Studies have also indicated difficulties in written language expression in connection with ADHD (22)*.*

Gender disparities have been observed for both childhood and adult ADHD (23). In children, the prevalence of ADHD is 3–4 times higher in boys compared to girls (24), whereas in adult ADHD the male-to-female ratio is closer to 1.0–1.5 (23,25). The presentation of ADHD symptoms in childhood is characterized by a predominance of hyperactivity-impulsivity in boys and inattention in girls (1), whereas adult women more often suffer from emotional symptoms and affective, eating, and somatization disorders than do men (23,25).

Several studies have demonstrated a high prevalence of psychiatric diagnoses such as conduct disorder (CD), antisocial personality disorder (ASPD), substance use disorder (SUD), ADHD and learning disorder among mainly male prison inmates (26–28), and one recent study showed that approximately 50% of male inmates have current ADHD and related problems (29). At the time of the present investigation there were no studies dedicated specifically to female convicts.

### Aims of the study

The aims of the study were to survey the frequency and severity of self-reported ADHD-related symptoms, difficulties and suffering, general assessment of functioning (GAF), and reading and spelling difficulties in patients seeking psychiatric care for common mental problems, the majority of whom are females, and in female prisoners convicted of severe crimes, in comparison with a sample of the general population. Our hypothesis was that ADHD-related symptoms, associated co-morbidity, and reading and spelling difficulties are over-represented in out-patient psychiatry and in female prisoners.

## Materials and methods

Three groups of study participants (see below) were surveyed in parallel during the same time period using a questionnaire covering childhood and current symptoms of ADHD, disabilities, and co-morbidity to be filled out anonymously. Since no personal information on non-participants was obtained, no analyses of missing data could be performed. The study was approved by the Uppsala University Ethics Committee. Informed consent in writing was obtained from each participant.

### The general population sample

At the time of this investigation in 2003, Uppsala County consisted of 300,379 inhabitants. A sample of 1000 (equal numbers of men and women) between 18 and 55 years of age was randomly selected from the population registry by an independent company (SYSteam Health & Care Udac AB). The questionnaire was mailed to the selected persons, and they were reminded twice by mail after the initial request to participate.

Out of the 1000 questionnaires distributed by mail 529 were returned; 12 incompletely filled out questionnaires were excluded from analyses. Thus, the total number of participants included was 236 men and 281 women, representing a participation rate of 52% (517/1000). The general population sample was not asked about current mental disorders or on-going medication.

### The psychiatric out-patient sample

The target population consisted of adult psychiatric out-patients in Uppsala County, women being in majority, seeking care mainly for affective, anxiety, sleep, and personality disorders, i.e. common co-morbid disorders in adult ADHD. Patients with previously diagnosed alcohol and/or substance use disorders (SUD), known to have a high frequency of ADHD, and chronic psychoses were not considered for inclusion. Four hundred questionnaires were distributed among local teams serving separate catchment areas within Uppsala County, each team receiving a number of questionnaires proportional to their average monthly attendance rate. The teams were instructed to hand out a copy of the questionnaire to every Swedish-speaking patient between 18 and 55 years of age after information about the study had been given orally and in writing by the reception staff. Four hundred and sixty-eight (*n* = 468) patients were offered to participate in the study, of which 400 volunteered to take part. Out of the 400 questionnaires distributed 369 were returned, 20 questionnaires were excluded due to incomplete answers, leaving 77 men and 272 women for analysis, representing a participation rate of 75% (349/468).

The main complaints (multiple complaints allowed) for seeking psychiatric care among participants who volunteered to respond were consistent with the following diagnostic groups: affective disorders (45%), anxiety disorders (47%), sleep or eating disorders (14.1%), personality disorders (2.6%), various other disorders (14.1%), unknown (2%). Only two participants mentioned ADHD as their main complaint.

A question about on-going medication was answered by 340 patients. The reported drugs were: selective serotonin re-uptake inhibitors (42.9%), other antidepressants (24.3%), anxiolytics (19.4%), antipsychotic drugs (6.8%), mood stabilizers (5%), hypnotics (21.2%), other psychoactive drugs and analgesics (12.9%), and medication for somatic disorders (12.6%). Only one participant was prescribed central stimulants.

### Female prison inmate sample

The Hinseberg prison is the largest prison for women in Sweden with the highest security level and is intended for female convicts from the whole country sentenced to long-term (6 years on average) imprisonment. The two most common crimes of conviction were severe drug crimes and murder/manslaughter—44% and 21%, respectively. Of the study group 57% (37/65) were Swedish, 5% (3/65) were citizens of other Nordic countries, and 38% (25/65) were citizens of other foreign countries (participants not speaking Swedish were assisted by interpreters). At the time of the study the number of inmates was 104; 65 high-security internees were available for the study and were invited to participate. Excluded from participation were: 17 inmates who had been transferred to a special unit for treatment of alcohol and drug addiction; 18 internees being transferred to an open-wing section since they were convicted of minor crimes; 4 being admitted to hospital. A total of 50 participants gave informed consent and, thus, the overall participation rate was 77% (50/65). Drugs for treatment of somatic disorders were prescribed to 18% of the female inmates, and 20% were prescribed antidepressants, antipsychotics, or hypnotics. None were treated with central stimulants or atomoxetine for ADHD.

### The questionnaire

The first part of the questionnaire covered the 18 symptoms of ADHD according to DSM-IV. Each question was supplemented by a short description of possible adult expressions of the symptoms. The response format was four-fold, depending on current presence and burden of symptoms of ADHD: never/seldom, sometimes, often, and very often. Each answer corresponded to a score from 0 to 3, giving the questionnaire a maximum score of 54 and a maximum total score of 27 for hyperactivity-impulsivity and inattention, respectively. A score of 2 or more of at least six out of nine symptoms of inattention and/or six out of nine symptoms of hyperactivity-impulsivity was required to be categorized as inattentive and/or hyperactive types of adult ADHD. Questions about ADHD symptoms during childhood as reported to the participant by parents or other informants were asked separately.

The second part of the questionnaire included questions on age and sex, reading and spelling difficulties (Yes/No answers). Functional impairment was assessed by asking the participants to rate difficulties and suffering caused by the current ADHD-related symptoms, using two 100-mm visual analogue scales (VAS) (end points: no difficulties/no suffering to totally handicapped) as well as a request to self-rate their global assessment of functioning (GAF)—according to DSM-IV—during the last year and the last two weeks (30,31). The psychiatric patients were also asked about reasons for seeking care and on-going medication. The prisoners were screened for conduct disorder (CD) and antisocial personality disorder (ASPD) by using relevant subscales of the self-report version of the DSM-IV and ICD-10 personality questionnaire (DIP-Q) (32,33). The time to fill out the questionnaire was approximately 20 minutes.

### Classification of groups and subgroups

Participants from the general population and out-patient psychiatry samples were divided into two main groups, ‘Hyperactive’ and ‘Not hyperactive’. Hyperactive participants were those who endorsed current ADHD symptoms (a score of 2 or 3 on at least six out of nine symptoms of hyperactivity-impulsivity) and hyperactivity-impulsivity in childhood. The hyperactive group was split into three subgroups. ‘Childhood only’ was fulfilled if the patient confirmed a history of hyperactivity in kindergarten, preschool, or primary school without endorsement of symptoms of adult hyperactivity-impulsivity. ‘Childhood and Adult’ was fulfilled if a history of childhood hyperactivity was reported and a rating of 2 or 3 on six or more DSM-IV symptoms of hyperactivity-impulsivity was endorsed in the questionnaire. ‘Adult only’ was fulfilled if six or more DSM-IV symptoms of hyperactivity-impulsivity were rated 2 or 3 and no report of childhood hyperactivity. The not hyperactive group, serving as a comparison group, consisted of participants not reporting childhood hyperactivity and with less than six DSM-IV symptoms of hyperactivity-impulsivity rated 2 or 3 according to the questionnaire.

The female prison inmate participants reporting childhood and/or adult ADHD symptoms of hyperactivity-impulsivity and/or inattention constituted the ‘ADHD group’. Participants without childhood symptoms and not fulfilling the criteria of a score of 2 or more on at least six out of nine symptoms of inattention or hyperactivity-impulsivity constituted the ‘Non-ADHD group’ and served as a comparison group. Each group consisted of 25 participants.

### Statistics

Data were analysed using SPSS, version 12.0.1. The chi-square test or Fisher's exact test was used to test for significant differences in frequencies between groups. Since symptom and GAF scores were not normally distributed, differences in mean scores were analysed using the Mann-Whitney U-test. Confounding by skewed gender proportions and higher prevalence of hyperactivity in the out-patient sample was corrected for by Mantel-Haenszel statistics. *P*-values of less than 0.05 were considered statistically significant.

## Results

### Age and sex

The mean (± SD) age of the participants was 33 ± 10 (median 32, range 18–55) years in the out-patient sample and 37 ± 11 (median 37, range 18–55) years in the general population sample (*P* < 0.001). There was no statistically significant difference in age between females and males in the two samples. The mean age of the prisoners was 35 ± 10 years (median 33.5, range 19–60).

The percentage of females in the general population sample was 54% and in the out-patient sample 78%. The prison inmates were all females.

### The general population and the out-patient psychiatry samples

#### Prevalence of hyperactivity-impulsivity

As shown in Table I, 32.4% of the participants in the out-patient psychiatry sample belonged to the hyperactive group versus 15.1% in the general population sample. The three subgroups, childhood only, childhood and adult, and adult only were more prevalent in the out-patient sample as compared to the general population sample (*P* < 0.01, *P* < 0.001, and *P* < 0.0001, respectively). The prevalence of adult and childhood hyperactivity-impulsivity was approximately three times higher among participants in out-patient psychiatry as compared to the general population (6.6% versus 2.1%). The prevalence of hyperactivity-impulsivity was higher in men than in women, 19.5% versus 11.4% (ratio 1.7) in the general population (*P* < 0.01), and 45.5% versus 27.7% (ratio 1.6) in out-patient psychiatry (*P* < 0.05).

**Table I.** Percentages (%) and number (*n*) of individuals categorized as Hyperactive (sum of the subgroups Childhood only, Childhood and Adult, Adult only) and Not hyperactive in the general population and out-patient psychiatry samples, respectively. For definitions of groups and subgroups, see Methods.

	General population			Out-patient psychiatry		
Groups and subgroups	Total % (*n*)	Men % (*n*)	Women % (*n*)	Total % (*n*)	Men % (*n*)	Women % (*n*)
*Hyperactive*	15.1 (78)	19.5 (46)	11.4 (32)	32.4 (113)^b^	45.5 (35)	28.7 (78)
Childhood only	11.8 (61)	16.1 (38)	8.2 (23)	16.6 (58)^a^	27.3 (21)	13.6 (37)
Childhood and Adult	2.1 (11)	1.8 (3)	2.8 (8)	6.6 (23)^b^	9.1 (7)	5.9 (16)
Adult only	1.2 (6)	2.1 (5)	0.4 (1)	9.2 (32)^b^	9.1 (7)	9.2 (25)
*Not hyperactive*	84.9 (439)	80.5 (190)	88.6 (249)	67.6 (236)	54.5 (42)	71.3 (194)
*Total*	100 (517)	100 (236)	100 (281)	100 (349)	100 (77)	100 (272)

The distribution of participants in the different subgroups was significantly different between men and women; chi-square *P* < 0.01 (general population), *P* < 0.05 (out-patients) in spite of the skewed sex-ratio in the out-patient sample as a confounding variable; Mantel-Haenszel test. ^a^*P*-value < 0.01, ^b^*P*-value <0.001, versus general population; chi-square test.

#### Symptom scores

Table II shows the total scores, the hyperactivity-impulsivity scores, and the inattention scores for the general population and out-patient psychiatry samples. The total scores were significantly higher in the out-patient psychiatry sample than in the general population sample for the hyperactive group (*P* < 0.001), the childhood only (*P* < 0.001), and the adult only (*P* < 0.01) subgroups, and the not hyperactive group (*P* < 0.001). Interestingly, the symptom scores were similar in the out-patient psychiatry and the general populations for the childhood and adult subgroup. There were no statistically significant differences in total symptom scores, inattention scores, or hyperactivity-impulsivity scores between men and women for the hyperactive groups in the two samples (data not shown).

**Table II.** Total symptom scores (mean ± SD), Inattention scores and Hyperactivity-Impulsivity (Hyp-Imp) scores in the different groups and subgroups in the general population and the out-patient psychiatry sample. Higher scores indicate higher degree of symptom severity. For definitions of groups and subgroups, see Methods.

	General population			Out-patient psychiatry		
Groups and subgroups	Total score (range 0–54)	Inattention (range 0–27)	Hyp-Imp (range 0–27)	Total score (range 0–54)	Inattention (range 0–27)	Hyp-Imp (range 0–27)
*Hyperactive*	18.0 ± 11.1^b^ (*n* = 78)	8.5 ± 5.7^b^	9.5 ± 6.3^b^	29.5 ± 10.0^b,d^ (*n* = 113)	15.3 ± 5.5^b^	14.3 ± 5.9^b^
Childhood only	13.5 ± 6.8^b^ (*n* = 61)	6.6 ± 4.2^b^	6.9 ± 3.7^b^	23.2 ± 8.5^b,d^ (*n* = 58)	13.3 ± 5.5^b^	9.9 ± 4.1^b^
Childhood and Adult	38.1 ± 6.7^b^ (*n* = 11)	17.8 ± 4.6^b^	20.3 ± 4.0^b^	35.4 ± 4.5^b^ (*n* = 23)	16.8 ± 4.1^b^	18.6 ± 2.4^b^
Adult only	27.0 ± 4.2^b^ (*n* = 6)	10.2 ± 3.9^a^	16.8 ± 3.0^b^	36.9 ± 7.4^b,c^ (*n* = 32)	17.8 ± 5.1^b^	19.1 ± 3.7^b^
*Not hyperactive*	10.4 ± 6.8 (*n* = 439)	5.7 ± 4.4	4.7 ± 3.5	16.1 ± 8.1^d^ (*n* = 236)	9.2 ± 5.1	6.9 ± 4.2
*Total*	11.5 ± 8.1 (*n* = 517)	6.1 ± 4.7	5.4 ± 4.4	20.4 ± 10.7 (*n* = 349)	11.2 ± 5.9	9.3 ± 5.9

^a^*P* < 0.05, ^b^*P* < 0.001, versus Not hyperactive; Mann-Whitney U-test. ^c^*P* < 0.01, ^d^*P* < 0.001, versus General population; Mann-Whitney U-test.

#### Difficulties and suffering attributed to ADHD-related symptoms

**Figure 1.**
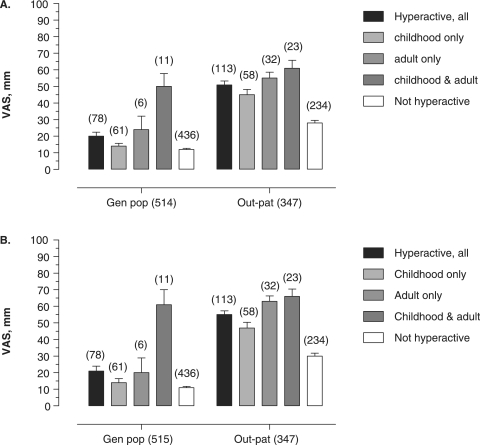
Difficulties (A) and Suffering (B) attributed to attention deficit hyperactivity disorder (ADHD)-related symptoms as assessed by the study participants on two separate visual analogue scales (VAS) (see Methods). The numbers of participants in different groups are given within brackets. The differences between Hyperactive versus Not hyperactive groups both for the general population (Gen pop) and the out-patient (Out-pat) samples are statistically significant (*P* < 0.001 in most cases). The differences are most pronounced for the group with both childhood and adult hyperactivity. The levels of difficulties and suffering in the out-patient sample were significantly higher (*P* < 0.001) than in the general population both for the Hyperactive and Not hyperactive group except for the subgroups with both childhood and adult symptoms.

As shown in Figure 1, the levels of difficulties and suffering due to ADHD-related symptoms in the out-patient sample were significantly higher both in the hyperactive group and in the not hyperactive group as compared to the general population sample (*P* < 0.001). Within the general population sample only the childhood and adult subgroup had significantly higher levels of both difficulties and suffering as compared to the not hyperactive group. In the out-patient sample all subgroups had significantly higher levels of both difficulties and suffering as compared to the not hyperactive group. There were no major differences between men and women within the two samples (data not shown).

#### Self-rated global assessment of function (GAF)

As shown in Table III, the GAF scores were significantly lower among participants in the out-patient sample, both during the past year and the past two weeks, as compared to the general population (*P* < 0.001). In the general population sample, only the childhood and adult subgroup had significantly lower GAF scores as compared to the not hyperactive group, both during the past year and the past two weeks (*P* < 0.01 and 0.001, respectively). The adult only subgroup had a significantly lower GAF score during the past two weeks as compared to the not hyperactive group (*P* < 0.05), whereas no difference was found with respect to the past year. The GAF scores for men and women did not differ significantly. In the out-patient psychiatry sample, the hyperactive group and all subgroups had significantly lower GAF scores than the not hyperactive group with the exception of the past two weeks for the childhood hyperactivity only subgroup. The GAF scores were not significantly different for men and women.

**Table III.** Self-rated general assessment of functioning (GAF) scores (range 1–100, where a score of 1 corresponds to inability to care for oneself, 50 to severe mental problems, and 100 to full mental health) over the past year and past week in the general population and out-patient psychiatry samples and broken down into the different subgroups. For definitions of groups and subgroups, see Methods.

	General population			Out-patient psychiatry		
Groups and subgroups	Total mean ± SD (*n*)	Men mean ± SD (*n*)	Women mean ± SD (*n*)	Total mean ± SD (*n*)	Men mean ± SD (*n*)	Women mean ± SD (*n*)
*Hyperactive*						
past year	83 ± 14 (75)	83 ± 15 (45)	83 ± 14 (30)	52 ± 12 (106)^c, d^	54 ± 12 (33)	52 ± 12 (73)
past two weeks	83 ± 14 (74)^a^	85 ± 13 (44)	80 ± 15 (30)	60 ± 15 (106)^a, d^	59 ± 19 (31)	60 ± 13 (75)
*Childhood only*						
past year	86 ± 13 (59)	84 ± 15 (37)	89 ± 9 (22)	54 ± 11 (54)^b^	57 ± 9 (20)	52 ± 12 (34)
past two weeks	86 ± 12 (58)	87 ± 12 (36)	85 ± 12 (22)	64 ± 14 (53)	64 ± 16 (18)	64 ± 13 (35)
*Childhood and Adult*						
past year	71 ± 15 (10)^b^	87 ± 6 (3)	64 ± 13 (7)	48 ± 13 (21)^c^	42 ± 16 (6)	50 ± 12 (15)
past two weeks	66 ± 14 (10)^c^	77 ± 15 (3)	61 ± 11 (7)	52 ± 17 (22)^c^	36 ± 17 (6)	58 ± 13 (16)
*Adult only*						
past year	75 ± 17 (6)	72 ± 17 (5)	90 (1)	53 ± 10 (31)^c^	54 ± 8 (7)	53 ± 11 (24)
past two weeks	75 ± 13 (6)^a^	74 ± 14 (5)	80 (1)	56 ± 11 (31)^c^	63 ± 13 (7)	54 ± 10 (24)
*Not hyperactive*						
past year	85 ± 14 (434)	88 ± 12 (189)	83 ± 15 (245)	61 ± 17 (225)^d^	62 ± 18 (41)	60 ± 17 (184)
past two weeks	86 ± 13 (428)	88 ± 13 (187)	85 ± 14 (241)	64 ± 17 (219)^d^	63 ± 18 (41)	65 ± 16 (178)
*Total*						
past year	85 ± 14 (509)	87 ± 13 (234)	83 ± 15 (275)	58 ± 16 (331)^d^	58 ± 16 (74)	58 ± 16 (257)
past two weeks	86 ± 14 (502)	87 ± 13 (231)	84 ± 14 (271)	63 ± 16 (325)^d^	61 ± 18 (72)	63 ± 15 (253)
*Missing information*						
past year *n* (%)	8/517 (1.5)	2/236 (0.8)	6/281 (2.1)	18/349 (5.2)	3/77 (3.9)	15/272 (5.5)
past two weeks n (%)	15/517 (2.9)	5/236 (2.1)	10/281 (3.6)	24/349 (6.9)	5/77 (6.5)	19/272 (7.0)

^a^*P*-value < 0.05, ^b^*P*-value < 0.01, ^c^*P*-value < 0.001 versus Not hyperactive; Mann-Whitney U-test. ^d^*P*-value <0.001, versus General population; Mann-Whitney U-test.

#### Reading and spelling difficulties

There was a significantly higher prevalence of reading and spelling difficulties in the out-patient sample than in the general population sample (*P* < 0.001 and 0.01, respectively); see Table IV. Interestingly, this was the case also for the not hyperactive group (*P* < 0.001 and 0.05, respectively). The frequencies of spelling difficulties in the hyperactive group were similar in both samples, whereas reading difficulties were more frequent in the out-patient sample (*P* < 0.05). For the childhood and adult subgroups the frequencies of reading and spelling difficulties were similar between the two samples. In the general population sample the subgroups with childhood hyperactivity as well as childhood and adult hyperactivity had significantly higher frequencies of both reading and spelling difficulties than did the not hyperactive group (*P* < 0.001 and < 0.001 respectively). There were no statistically significant differences between men and women in the two samples. In the out-patient psychiatry sample most hyperactivity subgroups were found to have higher frequencies of reading and spelling difficulties than did the not hyperactive group. The percentages in women were similar to those in men.

**Table IV.** Percentages (proportions) with reading and spelling difficulties within the different groups and subgroups in the general population and the out-patient psychiatry samples. For definition of groups and subgroups, see Methods.

	General population			Out-patient psychiatry		
Categories and subgroups	Total % (*n/N*)	Men % (*n/N*)	Women % (*n/N*)	Total % (*n/N*)	Men % (*n/N*)	Women % (*n/N*)
*Hyperactive*						
reading difficulties	23.1 (18/78)^c^	21.7 (10/46)	25.0 (8/32)	38.9 (44/113)^c,d^	42.9 (15/35)	37.2 (29/78)
spelling difficulties	30.8 (24/78)^c^	26.1 (12/46)	37.5 (12/32)	24.8 (33/133)^c^	28.6 (10/35)	29.5 (23/78)
*Childhood only*						
reading difficulties	16.4 (10/61)^a^	15.8 (6/38)	17.4 (4/23)	37.9 (22/58)^b^	33.3 (7/21)	40.5 (15/37)
spelling difficulties	24.6 (15/61)^c^	21.0 (8/38)	30.4 (7/23)	32.8 (19/58)^c^	33.3 (7/21)	32.4 (12/37)
*Childhood and Adult*						
reading difficulties	54.5 (6/11)^c^	66.7 (2/3)	50.0 (4/8)	44.0 (11/23)^b^	57.1 (4/7)	43.8 (7/16)
spelling difficulties	63.6 (7/11)^c^	66.7 (2/3)	62.5 (5/8)	43.5 (10/23)^b^	42.9 (3/7)	43.8 (7/16)
*Adult only*						
reading difficulties	33.3 (2/6)	40.0 (2/5)	(0/1)	34.4 (11/32)^a^	57.1 (4/7)	28.0 (7/25)
spelling difficulties	33.3 (2/6)	40.0 (2/5)	0.0 (0/1)	12.5 (4/32)	0.0 (0/7)	16.0 (4/25)
*Not hyperactive*						
reading difficulties	6.8 (30/439)	8.4 (16/190)	5.6 (14/249)	17.8 (42/236)^f^	23.8(10/42)	16.5 (32/194)
spelling difficulties	7.5 (33/438^g^)	11.1(21/189^g^)	4.8 (12/249)	13.1 (31/236)^d^	14.3 (6/42)	12.9 (25/194)
*Total*						
reading difficulties	9.3 (48/517)	11.0 (26/236)	7.8 (22/281)	24.6 (86/349)^f^	32.5 (25/77)	22.4 (61/272)
spelling difficulties	11.0 (57/516^g^)	14.0 (33/235^g^)	8.5 (24/281)	18.3 (64/349)^e^	20.8 (16/77)	17.6 (48/272)

^a^*P*-value < 0.05, ^b^*P*-value < 0.01, ^c^*P*-value < 0.001 versus Not hyperactive; chi-square test. ^d^*P*-value < 0.05, ^e^*P*-value < 0.01, ^f^*P*-value < 0.001, versus general population, chi-square test. Confirmed by Mantel-Haenszel's test due to a higher degree of hyperactivity as a confounding variable in the out-patient sample. ^g^One participant with missing value.

### Female prison inmate sample

A total of 50% of the female inmates reported symptoms of ADHD in childhood and/or as adults; 6 of the 50 study participants (12%) reported inattention only in childhood. Fifteen (30%) had both childhood inattention and adult symptoms of ADHD. Seven of those endorsed a rating of 2 on six or more symptoms of inattention matching the inattentive type of ADHD, and another seven endorsed a rating of 2 on six or more symptoms of both inattention and hyperactivity-impulsivity matching the combined type of ADHD. One participant endorsed a rating of 2 on six or more symptoms of hyperactivity-impulsivity but not inattention, thus matching the hyperactivity-impulsivity type of ADHD. Four participants (8%) endorsed current ADHD symptoms (i.e. only as adults), one having a rating of 2 on six or more symptoms of inattention, another on six or more symptoms of hyperactivity-impulsivity, and two on six or more symptoms of both inattention and hyperactivity-impulsivity.

The symptom scores of ADHD, difficulties and suffering attributed to symptoms of ADHD, global assessment of functioning (GAF), and reading and spelling difficulties were compared between the 25 participants in the ‘ADHD group’ and the 25 participants in the ‘non-ADHD group’. As shown in Table V, the ADHD group had statistically significantly higher inattention scores, hyperactivity-impulsivity scores, and total scores than did the non-ADHD group. Total symptom scores and inattention scores were also found to be statistically significantly higher in the ADHD group with ASPD than without (*P* < 0.05; Mann-Whitney U-test). Difficulties and suffering attributed to symptoms of ADHD, the GAF ratings for the past year and for the past two weeks, and reading and spelling difficulties were also statistically significantly lower in the ADHD group than in the non-ADHD group.

**Table V.** Inattention and hyperactivity-impulsivity scores, difficulties and suffering attributed to attention deficit hyperactivity disorder (ADHD)-related symptoms, global assessment of functioning (GAF), frequency of reading and spelling difficulties, and frequency of conduct (CD) and antisocial personality disorder (ASPD) in the ADHD and the non-ADHD groups (see text).

	ADHD group (*n* = 25)	Non-ADHD group (*n* = 25)	*P*-value
Total scores	33.2 ± 7.5	11.8 ± 5.6	*P* < 0.001^a^
Inattention scores	17.5 ± 4.4	6.0 ± 3.7	*P* < 0.001^a^
Hyperactivity-Impulsivity scores	15.7 ± 4.9	5.9 ± 3.3	*P* < 0.001^a^
Difficulties	52 ± 25	19 ± 24	*P* < 0.001^a^
Suffering	52 ± 25	18 ± 18	*P* < 0.001^a^
GAF past year^c^	62 ± 17	81 ± 17	*P* < 0.001^a^
GAF past two weeks^c^	66 ± 18	79 ± 16	*P* < 0.01^a^
Reading difficulties	36% (9/25)	8% (2/25)	*P* < 0.05^b^
Spelling difficulties	32% (8/25)	8% (2/25)	ns
CD	84% (21/25)	28% (7/25)	*P* < 0.001^b^
ASPD	76% (19/25)	20% (5/25)	*P* < 0.001^b^

^a^Mann-Whitney U-test; ^b^Fisher's exact test. ^c^*n* = 24 in each group.

The frequencies of inmates fulfilling the criteria for conduct disorder (CD), antisocial personality disorder (ASPD), and adult ASPD without a childhood history of CD according to the DIP-Q self-report were statistically significantly more common in the ADHD group versus the non-ADHD group (*P* < 0.001; chi-square test).

## Discussion

The main findings in the present study are the high prevalence of ADHD-related symptoms associated with increased difficulties and suffering, lower GAF scores, and increased frequencies of reading and spelling difficulties in out-patient psychiatry, in females as well as in males, and in female convicts as compared with the general population.

In the general population sample 15.1% were categorized as hyperactive with high scores of inattention and hyperactivity-impulsivity and increased levels of difficulties and suffering attributed to these symptoms relative to the not hyperactive group. The childhood and adult subgroup in the general population sample had higher total rating scale scores, more difficulties and suffering attributed to ADHD-related symptoms, lower GAF scores, and more reading and spelling difficulties than did the other subgroups. The size of this group (2.1%) is within the range of published prevalence figures of clinically diagnosed adult ADHD (cf. Introduction).

The main finding in psychiatric out-patients was that the hyperactive group was twice as large compared to the general population. The subgroup with ADHD-related symptoms of hyperactivity-impulsivity in combination with a childhood history of hyperactivity was three times higher in the out-patient psychiatry sample mainly suffering from mood and anxiety disorders than in the general population (6.6% versus 2.1%). Almeida Montes et al. (34) comparing an out-patient with a non-patient sample also reported a three times higher prevalence among the out-patients, and a high rate of ADHD has been found in general psychiatry out-patients by Nylander et al. 2009 (13).

The out-patient psychiatry sample had higher symptom scores (both for hyperactivity-impulsivity and inattention) than did the general population and higher ratings of difficulties and suffering attributed to ADHD-related symptoms. Self-rated GAF scores were lower within out-patient psychiatry than in the general population, both in the hyperactive and the not hyperactive group. There was also a higher prevalence of reading and spelling difficulties among out-patients as compared with the general population, both for the hyperactive and the not hyperactive group.

Interestingly, only two participants in the out-patient psychiatry group were seeking care because of symptoms of ADHD, and only one received treatment for ADHD despite histories of childhood hyperactivity-impulsivity and the presence of ADHD-related symptoms.

Hyperactivity-impulsivity was more prevalent in men than in women in both samples with a male-to-female ratio of 1.6–1.7, which is commensurate with previous reports (23). However, there were no major sex differences regarding rating scale scores, difficulties and suffering attributed to ADHD-related symptoms, GAF scores, or reading and spelling difficulties in the hyperactive groups in the two samples. There was a majority of women in the present out-patient psychiatry sample, which might be an effect of a patient selection mechanism. Most participants were seeking care mainly for affective and anxiety disorders, which are more prevalent in women. Reading and spelling difficulties within out-patient psychiatry, especially among individuals reporting childhood hyperactivity with or without persistence into adulthood, is consistent with previous reports in connection with ADHD (21,22). Interestingly, reading and spelling difficulties, especially in the hyperactive groups, were similar in the two samples. The overall high prevalence of reading difficulties in out-patient psychiatry, even among participants with low ratings of ADHD-related symptoms, was not expected. This finding requires attention in clinical practice.

The female prison inmates belonging to the ‘ADHD group’, comprising 50% of the participants, all reported childhood inattention and were characterized by higher rating scale scores, higher levels of difficulties and suffering attributed to ADHD-related symptoms, lower GAF scores, more reading and spelling difficulties, and higher frequencies of conduct disorder (CD) and current antisocial personality disorders (ASPD), as compared with the ‘non-ADHD group’. Thus, there seems to be a high degree of co-occurrence of childhood inattention, CD and ASPD, and ADHD-related problems in female convicts. In all rating measures the ‘ADHD group’ differed from the general population sample and had similar ratings and frequencies to the hyperactivity group in the out-patient psychiatry sample. The ‘non-ADHD group’ had similar scores and frequencies as the not hyperactive group in the general population sample.

The literature on ADHD in female offenders has recently been reviewed in a study by Rosler et al. (35). They reported that 10% of 110 incarcerated women aged 34 ± 12 years had a clinical diagnosis of ADHD versus 45% of incarcerated men aged 19.5 ± 2 years. Studies cited by Rosler et al. (35) reported percentages of ADHD in female offenders that varied between 6% and 68%. Although not formally diagnosed, our study of women convicted for severe crimes indicated that up to 50% might qualify for a clinical diagnosis of ADHD.

The participation rate among patients within out-patient psychiatry was 75%, and 77% in the prisoners. Although the participation rate among randomly selected participants in the general population was lower, 52%, it was still acceptable compared to similar studies in the literature (6,8).

There are certain limitations of the present study. Results are based on surveys of self- reported symptoms and not on clinical evaluations. However, the aim of the study was to substantiate the impression that adult ADHD is under-recognized in clinical practice. The robust findings of the study support our hypothesis that ADHD and related problems are over-represented in psychiatry and emphasize the need for proper clinical studies and improved assessments of patients in clinical practice in general. In order to secure honest answers and a high participation rate we chose to perform this survey anonymously, which, however, precluded analyses of missing data. Also, ADHD-related symptoms in combination with reading difficulties might be more frequent among non-participants leading to a falsely low prevalence estimate of ADHD-related symptoms in the samples under study. The low number of participants in some subgroups precluded detailed statistical analyses. The ADHD rating scale used in the present study was constructed on the basis of the DSM-IV criteria for ADHD. The questions of the present scale are similar or almost identical to the later published official WHO rating scale to evaluate adult ADHD: the Adult ADHD Self-Report Scale (ASRS) (36–38), which was not available when the present study was initiated. The major difference between the present scale and ASRS is that it has a four-step response format (0 to 3). A criterion was considered to be fulfilled if the respondent scored 2 or 3, corresponding to ‘often’ or ‘very often’. ASRS has a weighted five-step symptom frequency score (0 to 4), where a positive outcome is categorized with a variation of a score of 2, 3, or more on each symptom corresponding to ‘sometimes’, ‘often’, and ‘very often’ (36–38).

Patients and convicts with known alcohol or substance use disorders, known to be associated with high prevalence rates of ADHD and related problems, were not included in the present surveys. This might deflate the prevalence rates observed. The advantage, however, is that it could be shown that the frequency ADHD and related problems among non-addicts are common and need to be acknowledged.

## Conclusions

The present study indicates that there is a high prevalence of adult ADHD both in general psychiatry and in convicts. In addition, the high prevalence and severity of ADHD-related symptoms and problems in women is highlighted. This prompts further clinical investigations using presently available assessment instruments and physician-administered rating scales in order to confirm and extend the present findings. Our results are consistent with the emerging literature on adult ADHD, a neglected issue in psychiatry and in the rehabilitation of convicts.

## References

[1] (2000).

[2] World Health Organization (1992). The ICD-10 classification of mental and behavioural disorders: clinical descriptions and diagnostic guidelines.

[3] Polanczyk G, de Lima MS, Horta BL, Biederman J, Rohde LA (2007). The worldwide prevalence of ADHD: a systematic review and meta-regression analysis. Am J Psychiatry.

[4] Biederman J, Mick E, Faraone SV (2000). Age-dependent decline of symptoms of attention deficit hyperactivity disorder: impact of remission definition and symptom type. Am J Psychiatry.

[5] Kessler RC, Adler L, Barkley R, Biederman J, Conners CK, Demler O (2006). The prevalence and correlates of adult ADHD in the United States: results from the National Comorbidity Survey Replication. Am J Psychiatry.

[6] Faraone SV, Biederman J (2005). What is the prevalence of adult ADHD? Results of a population screen of 966 adults. J Atten Disord.

[7] Barkley RA, Murphy KR, Fischer M, ADHD in adults (2007). What the science says.

[8] Fayyad J, De Graaf R, Kessler R (2007). Cross-national prevalence and correlates of adult attention-deficit hyperactivity disorder. Br J Psychiatry.

[9] Davidson MA (2008). ADHD in adults: a review of the literature. J Atten Disord.

[10] Faraone SV, Biederman J, Spencer T, Mick E, Murray K, Petty C (2006). Diagnosing adult attention deficit hyperactivity disorder: are late onset and subthreshold diagnoses valid?. Am J Psychiatry.

[11] Barkley RA, Brown TE (2008). Unrecognized attention-deficit/hyperactivity disorder in adults presenting with other psychiatric disorders. CNS Spectr.

[12] Nylander L, Holmqvist M, Gustafson L, Gillberg C (2009). ADHD in adult psychiatry. Minimum rates and clinical presentation in general psychiatry outpatients. Nord J Psychiatry.

[13] Tamam L, Karakus G, Ozpoyraz N (2008). Comorbidity of adult attention-deficit hyperactivity disorder and bipolar disorder: prevalence and clinical correlates. Eur Arch Psychiatry Clin Neurosci.

[14] Ryden E, Thase ME, Straht D, Aberg-Wistedt A, Bejerot S, Landen M (2009). A history of childhood attention-deficit hyperactivity disorder (ADHD) impacts clinical outcome in adult bipolar patients regardless of current ADHD. Acta Psychiatr Scand.

[15] Barkley RA, Fischer M, Smallish L, Fletcher K (2004). Young adult follow-up of hyperactive children: antisocial activities and drug use. J Child Psychol Psychiatry.

[16] Weinstein D, Staffelbach D, Biaggio M (2000). Attention-deficit hyperactivity disorder in posttraumatic stress disorder: differential diagnosis in childhood sexual abuse. Clin Psychol Rev.

[17] Philipsen A, Limberger MF, Lieb K, Feige B, Kleindienst N, Ebner-Priemer U (2008). Attention-deficit hyperactivity disorder as a potentially aggravating factor in borderline personality disorder. Br J Psychiatry.

[18] Brook U, Boaz M (2005). Attention deficit and hyperactivity disorder (ADHD) and learning disabilities (LD): adolescents' perspective. Patient Educ Couns.

[19] Mayes SD, Calhoun SL, Crowell EW (2000). Learning disabilities and ADHD: overlapping spectrum disorders. J Learn Disabil.

[20] Dykman RA, Ackerman PT (1991). Attention deficit disorder and specific reading disability: separate but often overlapping disorders. J Learn Disabil.

[21] Rasmussen K, Almvik R, Levander S (2001). Attention deficit hyperactivity disorder, reading disability, and personality disorders in a prison population. J Am Acad Psychiatry Law.

[22] Stevenson J, Pennington BF, Gilger JW, DeFries JC, Gillis JJ (1993). Hyperactivity and spelling disability: testing for a shared genetic aetiology. J Child Psychol Psychiatry.

[23] Robison RJ, Reimherr FW, Marchant BK, Faraone SV, Adler LA, West SA (2008). Gender differences in 2 clinical trials of adults with attention-deficit/hyperactivity disorder: a retrospective data analysis. J Clin Psychiatry.

[24] Szatmari P, Offord DR, Boyle MH (1989). Ontario Child Health Study: prevalence of attention deficit disorder with hyperactivity. J Child Psychol Psychiatry.

[25] Rasmussen K, Levander S (2009). Untreated ADHD in adults: are there sex differences in symptoms, comorbidity, and impairment?. J Atten Disord.

[26] Vermeiren R, De Clippele A, Deboutte D (2000). A descriptive survey of Flemish delinquent adolescents. J Adolesc.

[27] Vermeiren R, De Clippele A, Deboutte D (2000). Eight month follow-up of delinquent adolescents: predictors of short-term outcome. Eur Arch Psychiatry Clin Neurosci.

[28] Einat T, Einat A (2008). Learning disabilities and delinquency: a study of Israeli prison inmates. Int J Offender Ther Comp Criminol.

[29] Rosler M, Retz W, Retz-Junginger P, Hengesch G, Scneider M, Supprian T (2004). Prevalence of attention deficit-/hyperactivity disorder (ADHD) and comorbid disorders in young male prison inmates. Eur Arch Psychiatry Clin Neurosci.

[30] Bodlund O, Kullgren G, Ekselius L, Lindstrom E, von Knorring L (1994). Axis V–-Global Assessment of Functioning Scale. Evaluation of a self-report version. Acta Psychiatr Scand.

[31] Ramirez A, Ekselius L, Ramklint M (2008). Axis V–-Global Assessment of Functioning scale (GAF), further evaluation of the self-report version. Eur Psychiatry.

[32] (1994).

[33] Bodlund O, Grann M, Ottosson H, Svanborg C (1998). Validation of the self-report questionnaire DIP-Q in diagnosing DSM-IV personality disorders: a comparison of three psychiatric samples. Acta Psychiatr Scand.

[34] Almeida Montes LG, Hernandez Garcia AO, Ricardo-Garcell J (2007). ADHD prevalence in adult outpatients with nonpsychotic psychiatric illnesses. J Atten Disord.

[35] Rosler M, Retz W, Yaqoobi K, Burg E, Retz-Junginger P (2009). Attention deficit/hyperactivity disorder in female offenders: prevalence, psychiatric comorbidity and psychosocial implications. Eur Arch Psychiatry Clin Neurosci.

[36] Kessler RC, Adler L, Ames M, Demler O, Faraone S, Hirpi E (2005). The World Health Organization Adult ADHD Self-Report Scale (ASRS): a short screening scale for use in the general population. Psychol Med.

[37] Adler LA, Spencer T, Faraone SV, Kessler RC, Howes MJ, Biederman J (2006). Validity of pilot Adult ADHD Self-Report Scale (ASRS) to rate adult ADHD symptoms. Ann Clin Psychiatry.

[38] Kessler RC, Adler LA, Gruber MJ, Sarawate CA, Spencer T, Van Brunt DL (2007). Validity of the World Health Organization Adult ADHD Self-Report Scale (ASRS) Screener in a representative sample of health plan members. Int J Methods Psychiatr Res.

